# Geographic monitoring for early disease detection (GeoMEDD)

**DOI:** 10.1038/s41598-020-78704-5

**Published:** 2020-12-10

**Authors:** Andrew Curtis, Jayakrishnan Ajayakumar, Jacqueline Curtis, Sarah Mihalik, Maulik Purohit, Zachary Scott, James Muisyo, James Labadorf, Sorapat Vijitakula, Justin Yax, Daniel W. Goldberg

**Affiliations:** 1 GIS Health & Hazards Lab, Department of Population and Quantitative Health Sciences, School of Medicine, Case Western Reserve University, Cleveland, OH USA; 2 University Hospitals Health System, Cleveland, OH USA; 3 GeoInnovation Service Center, Department of Geography, Texas A&M University, College Station, TX USA

**Keywords:** Diseases, Health care

## Abstract

Identifying emergent patterns of coronavirus disease 2019 (COVID-19) at the local level presents a geographic challenge. The need is not only to integrate multiple data streams from different sources, scales, and cadences, but to also identify meaningful spatial patterns in these data, especially in vulnerable settings where even small numbers and low rates are important to pinpoint for early intervention. This paper identifies a gap in current analytical approaches and presents a near-real time assessment of emergent disease that can be used to guide a local intervention strategy: Geographic Monitoring for Early Disease Detection (GeoMEDD). Through integration of a spatial database and two types of clustering algorithms, GeoMEDD uses incoming test data to provide multiple spatial and temporal perspectives on an ever changing disease landscape by connecting cases using different spatial and temporal thresholds. GeoMEDD has proven effective in revealing these different types of clusters, as well as the influencers and accelerators that give insight as to why a cluster exists where it does, and why it evolves, leading to the saving of lives through more timely and geographically targeted intervention.

## Introduction

Geographic aspects of coronavirus disease 2019 (COVID from this point forward) have been widely reported in popular and scientific media with maps visualizing areas experiencing the greatest intensity, patterns of risk, or characteristics of diffusion^1–6^. At the federal and state levels, county or zip code maps of positive tests, hospitalizations and mortality inform professionals and the public alike using spatially enhanced dashboards (e.g., CDC Covid Data Tracker: https://www.cdc.gov/covid-data-tracker/; Ohio Department of Health Covid-19 Dashboard: https://coronavirus.ohio.gov/wps/portal/gov/covid-19/dashboards/overview; Cleveland Confirmed and Probable COVID-19 Public Dashboard: http://www.clevelandhealth.org/). However, the map is not just a means of communicating geographic patterns across a country or region, it can also be used as a tool for local response. For example, locations of positive cases in conjunction with different types of congregate housing can give a vital first impression of where to mobilize resources at a building-specific scale for a hospital or county health department. While developing a spatial support system during the beginning stages of the pandemic, the authors realized that there was a need to refocus more traditional spatial epidemiology using a syndromic surveillance lens to leverage health system access to diverse data streams from different sources, and at different geographic scales and temporal cadences. Without such integration, there are missed opportunities for hospitals to mobilize early intervention activities and save lives (e.g., targeted community testing, hospital intercept teams going into skilled nursing facilities).

Responding to an epidemic presents spatial challenges beyond normal “spatial epidemiological” or “spatial science” research. Traditionally, for some forms of spatial analysis, disease data are aggregated by zip codes, counties, or other larger units to protect patient privacy while still being able to discern national or regional patterns. In other cases where the data are more granular and available to those who have permissions to view Protected Health Information (PHI), hot spot mapping is employed^7–9^, usually utilizing a GIS to tease out and visualize patterns in the surveillance. Such applications and advances have recently been made on COVID by several teams utilizing clustering and spatial modeling approaches at the county scale in the U.S.^10–13^. Such analytical methods continually evolve through conceptual advances such as incorporating context or developing new field methods along with the associated spatial software^14,15^. While still vitally important for understanding the geographic characteristics of disease, COVID presented a novel spatial health challenge that has not previously been faced. COVID is a geographically ubiquitous disease transmitting at a high rate, producing near real time data allowing for (and demanding) a continuous monitoring, analysis and understanding of the infection landscape. Every location is impacted, or has the potential to be impacted, and so insights and response are by definition, local. Test results (and associated variables such as test type), symptomology, associated medical factors of those infected and their background neighborhoods, as well as various built environment characteristics continuously flow to present a dynamic “big data” challenge. At the same time the “rules of engagement” in terms of how the disease spreads, who is most impacted, and what policy decisions would be the most effective to limit infection are continuously changing. At the beginning it was soon established that there were disproportionate impacts on certain vulnerable populations such as those that live in congregate housing, the elderly, and people with underlying comorbidities^16,17^. In the United States these vulnerabilities are often concentrated in minority populations resulting in racial health disparities^18^. From a hospital and health system perspective, such disease propagation can rapidly tax capacity and resources. From a humanitarian dimension, being able to liaise with local communities, municipality or even building managers requires both spatially and temporally granular insights. While there remain operational challenges such as uneven testing practices^18^, there are no mechanical reasons why positive COVID test results cannot be analyzed in real time as they flow into a health system which when combined with severity of the associated symptoms, details about the person (especially age) including previous medical histories of the patient, and the background neighborhood “risk” can all be added to provide a contextualized understanding of not only where disease occurs but why, and where it is likely to spread next.

More traditional spatial approaches to disease cluster detection are somewhat static in operation and underlying concept, such as the need for an appropriate denominator or attaching statistical significance to output findings. Note that Kulldorf’s (2005) space–time permutation scan statistic is not reliant on a reference population^27^. While these are still, of course, important, and the methods described in this paper should ideally work in parallel with more robust population based analyses, with syndromic surveillance interest lies in the *first* case in a post-acute care home, or the temporal pattern of emerging positives in an apartment complex, or along a rural street, or how houses on city streets “emerge” suggesting a local transmission mechanism. By identifying these emergent patterns in the continuous data streams available to health systems, intercept teams can be targeted to areas of concern with strategies (such as quarantine or facility lock downs) to reduce further transmission. As it was quickly established that this virus spreads effectively and to disastrous effect in congregate housing^19,20^, the time lag between knowing when a case emerges inside, or even proximate to the home, and when an intercept team can be mobilized can literally save lives. Simply put, there are not enough GIS analysts, in every location, to answer these questions in an ongoing timely fashion especially when we are in a disaster mode, with the whole operation becoming overwhelming from both a data flow and personal effectiveness perspective^21^. Here we present an alternative method to granular cluster detection that has proven to be able to stop spread, save lives, is transferable to different operations, is scalable and robust.

## Methods

### Operationalizing from data inflow to output designed for intervention

In order to operationalize the new form of clustering, different spatial data manipulations were required. As with most health systems, patient data includes a street address which has to be geocoded. Here a continuous feed of COVID-19 test results was fed through the health system’s enterprise geocoder. As the clustering described here is especially sensitive to not only geocoded accuracy but also precision, a two stage geocoding approach was used. The first geocoding process resulted in typically mapped objects along with different levels of success based not only on the actual geocode (the name or address used), but also the type of match (rooftop or polygon centroid). The second geocode, using a different software, focused on those addresses lacking the required precision^22^. After this two stage geocoding process, an automated set of queries were generated using a specially developed spatial database. S*entinel*, *micro* and *neighborhood* agglomerative and density based clusters (see the subsequent section for definitions) were calculated.

### Conceptualizing a spatial syndromic surveillance approach

Conceptually, for the novel cluster criteria, if X members connected to each other within Y distance meets a defined member threshold then a cluster is identified. These clusters are not static, but can grow when a new positive case is identified within Y distance of one of those cluster members. For example, for the *sentinel cluster*, here reported at 100 m and 2 members, if two positive cases (mapped by their residence) appear within 100 m of each other, then a *sentinel cluster* centroid is calculated. If another positive case falls within 100 m of either of these cluster members, then the cluster grows. This is repeated until no further members are added. A convex hull is automatically calculated using all the exterior members. This *sentinel cluster* is the first early warning, with results sometimes including members within the same household, living within the same congregate housing, or neighbors on the same or connecting street. While potentially the creation of these clusters could be continuous as is the incoming test result data, the reality of validating the geocoding, and then fully interpreting and prioritizing each cluster means that this happens at least once daily, or in response to other syndromic surveillance leading indicators, such as an increase in ambulance runs. The interpretation of findings includes whether the cluster coincides with obvious vulnerabilities, such as cases being inside a post-acute care home, or other forms of congregate housing, or being residential but proximate to either of those. The sentinel cluster provides an early warning for geographically targeted intervention, for example in the form of directing intercept teams. Another advantage of the sentinel cluster is that an overreliance on one address or name sometimes means cases are not correctly attributed to a post-acute care home location, the *sentinel cluster* helps remove this “fuzziness”. Geocoding accuracy and precision is, unfortunately, often a neglected aspect of spatial health research^28^. Using the Care Home example—a “campus” might include a variety of names, sometimes associated with different aspects of care. These buildings might also contain different addresses and apartments. The net result is the same general campus might have 10 or more addresses or combinations of address components, including the insertion of names and apartment identifiers. These can be corrected manually with sufficient attention, however the resulting coordinate in either the original or corrected geocode can vary considerably. The challenge is to have a tool precise enough to capture the singular risk posed by and to the campus, while not overly smoothing or aggregating positive cases over a larger area such as a census block group or neighborhood. Initially all positive test results were used in the formation of clusters but response “evolved” into a 21 day model based on developing medical insights into the disease and the need to include a “recovery logic” where the connection between positive cases was no longer thought to be epidemiologically connected. Including shorter more emergent time frames such as 7 and even 3 day clusters capture patterns of emergence, and these have now become the standard for the team’s syndromic surveillance as the response becomes more fine-tuned to targeted interventions. By having each cluster member’s medical information automatically joined, each cluster can also be characterized by, for example, its average age, co morbidity burden or severity of symptoms displayed. During the beginning of the epidemic, or for quieter phases, each sentinel cluster can be further investigated for underlying “driver” characteristics (such as being within specialized congregate living), while in periods of increased activity when hundreds of sentinel clusters are generated, the emergent versions (7 and 3 days) provide a quick visual assessment of an otherwise overwhelming disease landscape helping to develop a quick understanding of the situation and where to prioritize. This is especially true when these analyses are performed on a daily basis as comparisons to previous days’ outputs further reinforce emergent patterns.

**Figure 1:**
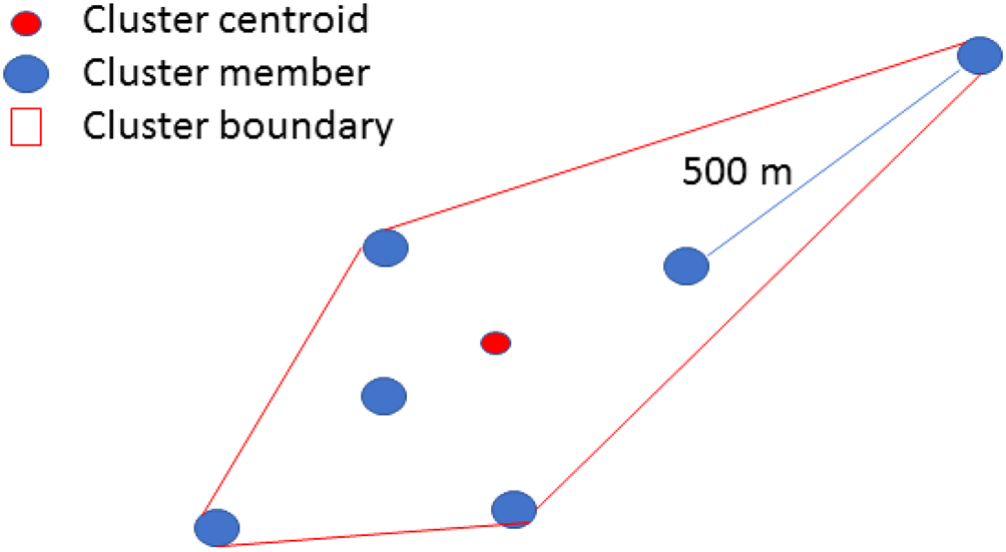
Example micro-cluster schematic.

The second type of signal, the *micro cluster*, uses the same conceptual logic but extends the distance to 500 m with a minimum membership of 5 cases (Fig. 1). The *micro cluster* provides an indication of either a more substantial congregate housing concern, such as in a public housing complex, or the indication of community spread where multiple positives, possibly outside the 100 m distance bands of the *sentinel cluster*, show that a section of a neighborhood has become “hot” due to a shared resource such as a grocery store.

Sometimes a high concentration of positive tests can result in a simultaneous new *sentinel* and *micro cluster*, for example in the same building complex where all members of a family test positive along with neighbors in a similar time frame. Alternatively, the complexity of the underlying diffusion pattern can be revealed if a single *sentinel cluster* continues to grow, or merges together with others. It is also possible for a *micro cluster* to contain no *sentinel clusters*. When used together, these clusters provide a vital insight into the granular drivers of an outbreak, and where spread is likely to happen next. For example, and with respect to the opening of universities, off campus housing has been found to generate both sentinel clusters (within the building) and micro clusters (due to multiple clusters being in close proximity).

The third signal, the *neighborhood cluster,* again uses the same logic but now captures the next level of the aggregation hierarchy to identify more widespread community activity. The *neighborhood cluster* has a distance bandwidth of 1000 m and a 10-member minimum. As with the *micro cluster*, this growth can reveal the various diffusions at play, possibly with *micro* and *sentinel clusters* merging, and with other positive case addresses not falling into either cluster type but “filling in” the geographic gaps between. For this cluster type, the automatic polygon creation around the exterior members provides a useful visual to be shared with health departments to target a region for specific intervention. By comparing this boundary extent over a series of days, the growth or contraction of community spread can be assessed, with vulnerable housing, the typical comorbidities of those falling inside, locations of pending tests awaiting results, building footprints, parcel and tax assessor information, aerial photography, even measures of social vulnerability all providing additional context as to potential risk and where intervention should be guided. In an example that has repeated multiple times in the Cleveland area, sentinel clusters have grown to form micro clusters which turn into neighborhood clusters containing multiple apartment complexes. Just as before, reading the interactions of the three cluster types, along with reference to Google Earth Imagery and Google Street View, revealed not only where cases were found, but where they were likely to spread to next. Those points of vulnerability, especially proximate post-acute care homes, would be identified and reported. While the *neighborhood cluster* superficially is the closest to more traditional clusters revealed through spatial analysis, it still differs as the underlying population has less importance compared to the amount of testing giving areal positivity. Indeed, this approach has been used to identify where overall testing numbers are lacking, and has been used to target outreach initiatives into these testing gaps. This is particularly relevant as seven months into the pandemic in Ohio, testing resources are still limited and aimed largely at severely symptomatic individuals.

For all these cluster types, the distance and minimum cluster membership can be varied to explore appropriate thresholds for different environments. For example, in rural environments physical distance between cases is greater, with neighbors along the same stretch of road being more readily identified using a 500 m micro cluster with 2 members. In this example all urban or dense residential areas are excluded as this is a “rural” *sentinel cluster*. Also, as previously mentioned, limiting the number of days allowable for a cluster to form can shift the focus to more emergent disease. While the three distances, number of members, and time frame of consideration have been decided upon through extensive testing for north east Ohio, and have been recommended for elsewhere, even in this region different variations have been used, especially with regards the more emergent shortened time frame, as the epidemic enters different phases. This flexibility is one of the explorative advantages of the technique, and it is expected that every region would vary these conditions based on local conditions. Achieving this flexibility is greatly benefitted by incorporating a spatial database and spatial programing which will be described in a subsequent section.

### Visualizing syndromic surveillance for a disaster response team

Flexible and insightful spatial investigations including various syndromic surveillance “triggers” are only as effective as the associated visual communication. During the response to any disaster, and especially to a pandemic such as this when everyone, everywhere is feasibly involved in surveillance, steps must be taken to automate decision support as much as possible in order to not experience analyst burnout. To achieve this a series of automatically generated output tables and maps are created. For example, output tables around key vulnerable institutions such as care homes are automatically generated to visualize changes in patterns across the cumulative totals for each day at different distance thresholds, with different guideline summary statistics added. In this way the rise in cases, and especially the presence of a first positive, can quickly be determined for these types of buildings. By adding in other distances around these facilities, for example 1000 or even 2500 m, the emerging proximate disease threat can also be established leading to pre-first case intervention. While not directly part of the cluster family described in this paper, the mechanics allowing for this type of spatial querying and display are the same.

**Figure 2:**
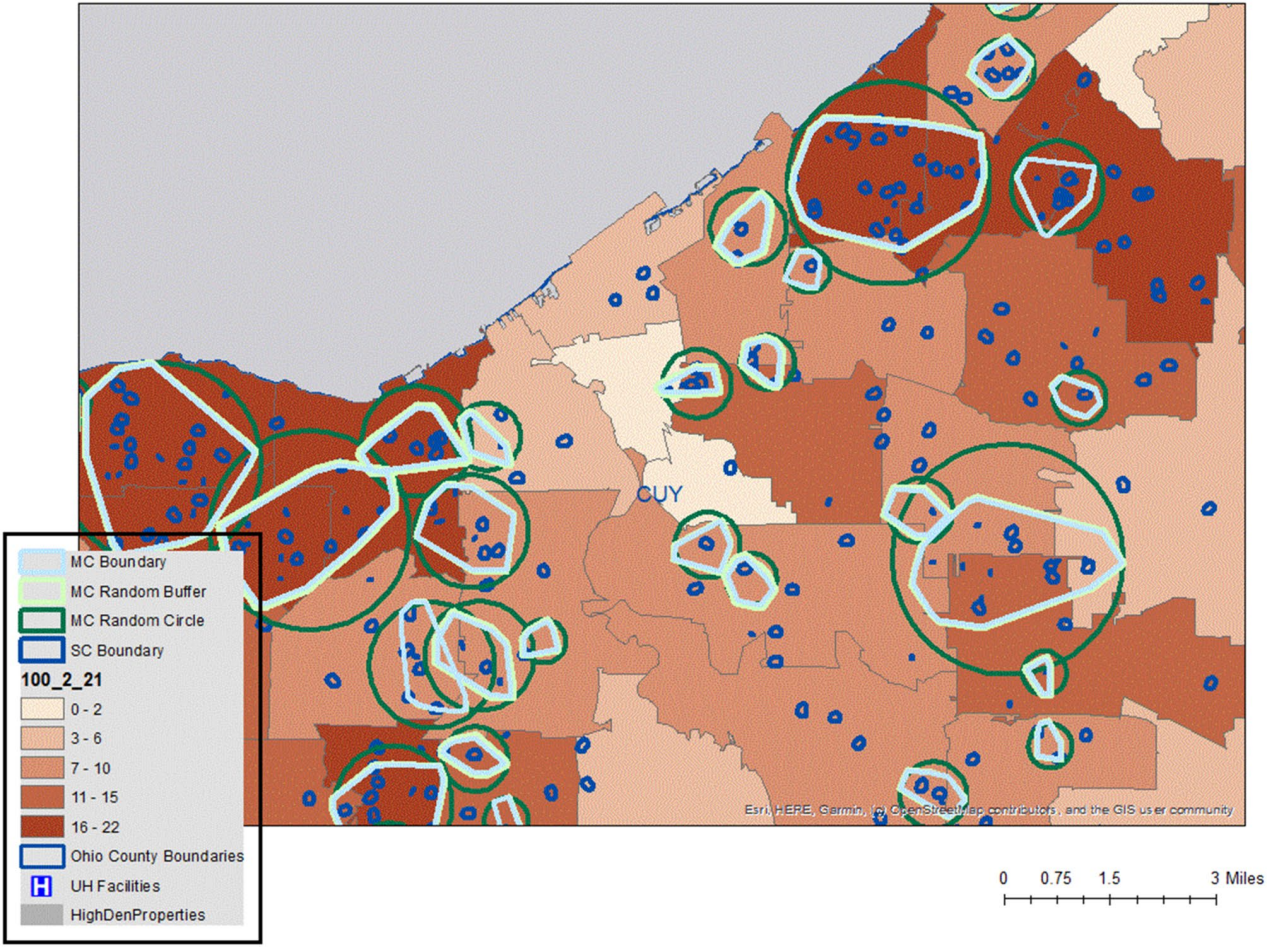
Example output showing micro and sentinel clusters with three different options of display dependent on the potential audience.

For the clusters, map boundaries were automatically generated using a convex hull polygon connecting all of the exterior cluster members, with a buffer polygon being used (with a radius set to the cluster type distance, such as 500 m) if all members reside at the same address. By producing the boundaries of the three cluster types in this way (or more if including the more emergent clustering) it is relatively easy to create hierarchical maps either in a Geographic Information System (GIS), or the visual display packages often preferred by health systems such as Tableau. Figure 2 displays a typical output using synthetic data for illustrative purposes only. Micro and Sentinel Clusters are displayed in their raw form, and also as slightly masked output with additional randomization around an exterior boundary and center point and as a count of clusters (here sentinel cluster count) for each polygon (here by school district). Having these different layers of output to mask the underlying granular disease situation is important for different audiences. For example, a hospital using its own data would want the most spatially specific output. A multi hospital collaboration would want an output that masked exactly where the patients resided but still require a high level of on-the-ground insight to help direct intercept teams, while a school district, only interested in whether children could return or not, need only be told how many clusters were in the neighborhood. These outputs can also be mapped onto any preferred underlay such as a street network, or social vulnerability layer.

The spatial database (described in the next section) is also used to automatically join each positive address to the patient’s past hospital record to establish the presence of existing co morbidities, and whether or not the person was admitted into hospital (being part of the hospital census meaning the symptoms were severe). This information could then be aggregated for each cluster. In this way it is easy to not only interpret the geographic pattern in Fig. 2 but also assess the average age structure of a cluster, whether the cluster members had co morbidities and whether outcomes required hospitalization. By also adding in negative test results, each cluster also receives an automatic measure of positivity for within its boundary.

### Spatial database development

**Figure 3:**
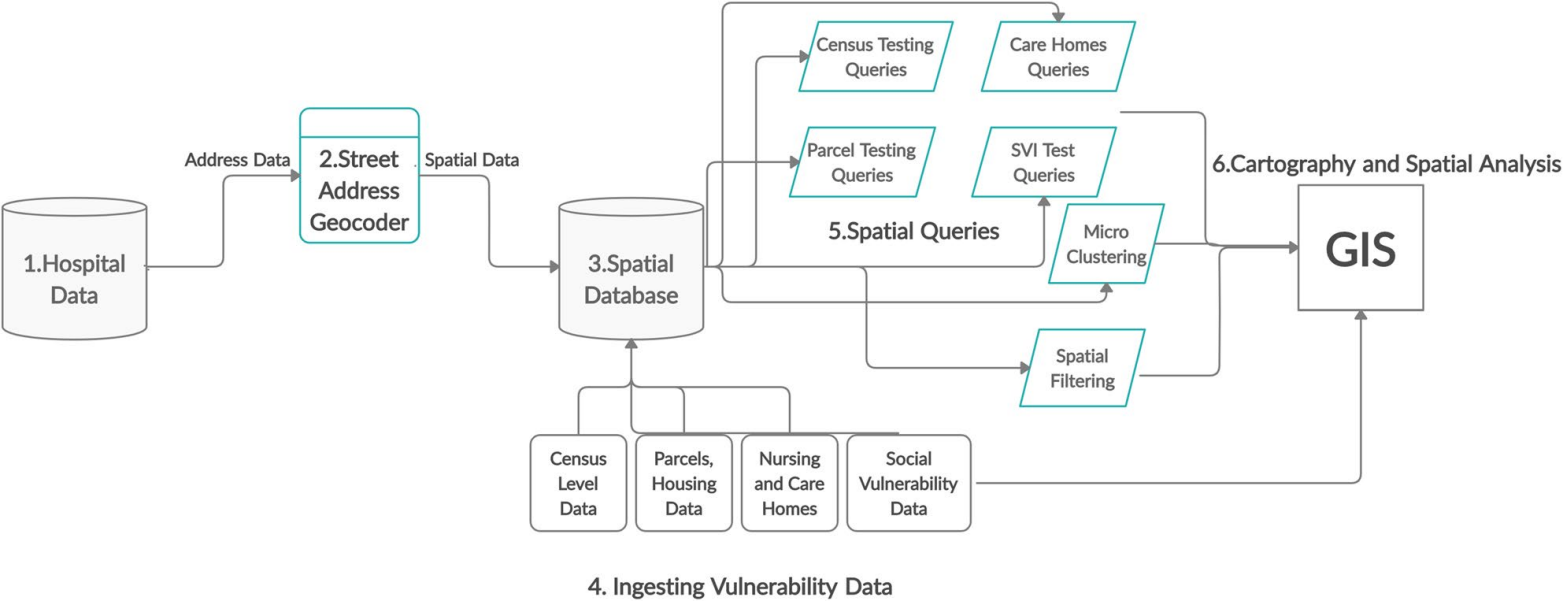
Schematic of the spatial database.

In order to achieve the previously described early warning systems and near real time clustering, including the various automatic querying, data combinations and tabular and mapped outputs, operational advances were required that went beyond typical hospital data system approaches. First, a spatial database was built that would ingest real time COVID test results from across the entire hospital system (Fig. 3). The spatial database ingests multiple health, emergency responder, social vulnerability data, and different congregate living spatial layers such as post-acute care homes, correctional facilities, and subsidized housing. While some congregate housing layers are available at the state level, such as correctional facilities, certain care home types, and HUD apartments, there are many congregate housing “gaps”, with availability (and quality) of these data varying by city and county.

More specifically, the spatial database was built using PostgreSQL, which is a free and open-source relational database management system (RDBMS) along with PostGIS which is an open source software program that adds support for geographical objects in PostgreSQL. Automatic batch jobs were written to pull locational data from the health system’s clinical and operational source systems, including COVID test data, encounter data from the Electronic Medical Record (EMR) system, Emergency Management Service (EMS) dispatches and previous addresses of those with Covid-related comorbidities that could be used to establish background neighborhood health risk. To contextualize the clinical data, various vulnerability measures commonly available such as those created by the Centers for Disease Control and Prevention (Social Vulnerability Index), and Health Resources & Service Administration (Area Deprivation Index) were also ingested and attached to the cluster outputs^23,24^.

While the clustering described here can also be coded without the use of a spatial database, much of the previously described flexibility in terms of modifying cluster characteristics, adding new queries, or combining other spatial data are far more efficient in such an environment. Two families of clustering types were utilized for the sentinel, micro and neighborhood clustering, agglomerative and a density based alternative. These were decided upon based on either conceptual ease (an important aspect in being able to convey how the clusters form), and then a more conservative approach which incorporates the concept of distance decay, that those members more tightly packed together have a greater influence on the cluster structure, which is also more efficient from a computing perspective.

### Agglomerative clustering

**Figure 4:**
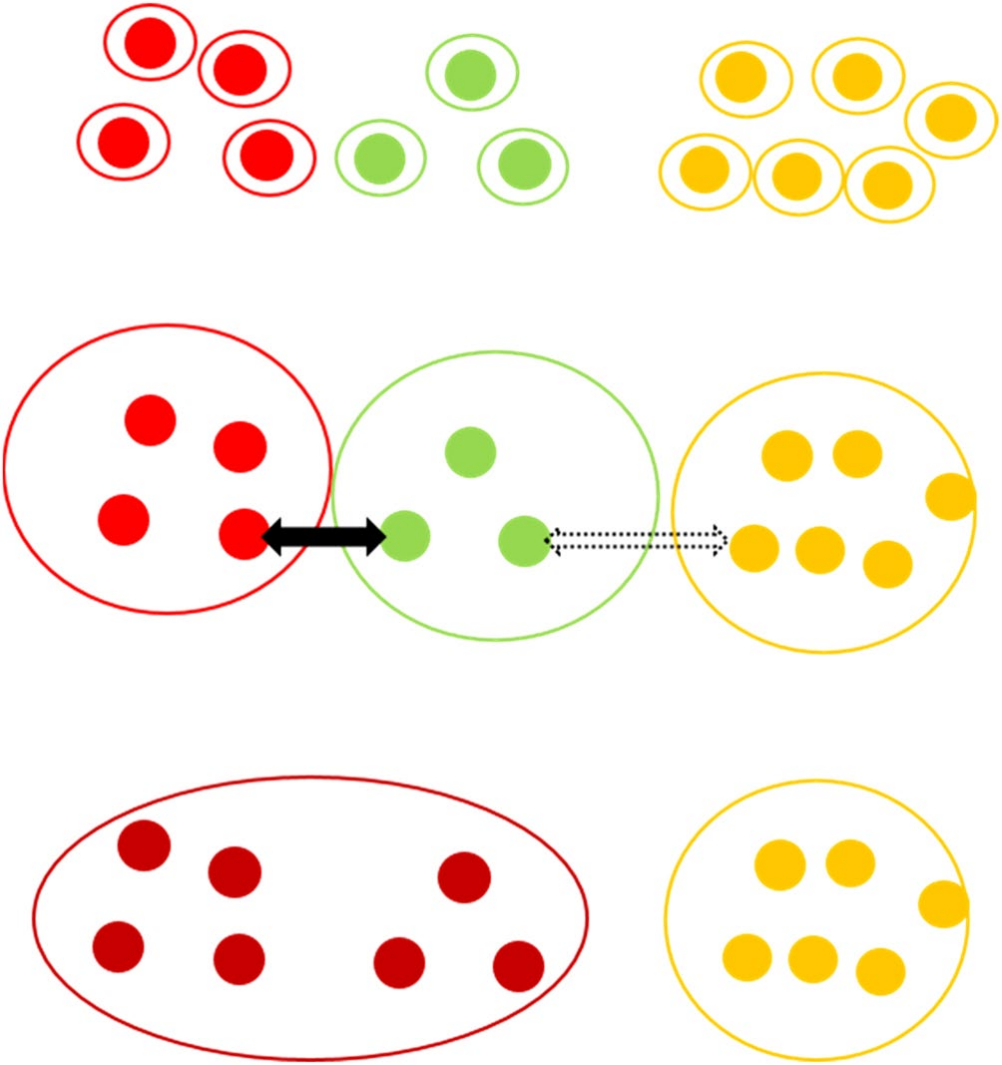
Demonstration of agglomerative clustering. The black arrow indicates a distance that is within the threshold distance (α), and the dashed arrow indicates a distance that is above the threshold.

Agglomerative Clustering is a type of hierarchical clustering that builds nested clusters by adding individuals through a bottom up approach starting with each observation point as a seed to which other seeds are added^25^. At the beginning, each observation is deemed as a cluster (Fig. 4). The merging between two seeds (in effect each being a “cluster”) is based on a single linkage criterion (Fig. 4 which minimizes the distance between the closest pairs of clusters (There are four different types of linkage criteria including Ward (minimizes the sum of squared differences within all clusters), complete linkage (minimizes the maximum distance between pairs of clusters), average linkage (minimizes the average of the distances between all pairs of clusters), and single linkage (minimizes the distance between the closest pairs of clusters).)), with a threshold distance (α) acting as a constraint to cluster merging. Clusters can grow (merge together) until the threshold is reached. The algorithm terminates after all points have been accounted for.

### Density-based spatial clustering of applications with noise (DBSCAN)

**Figure 5:**
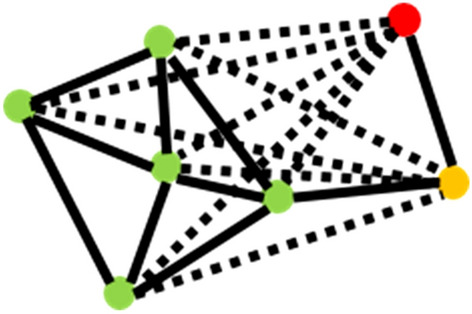
DBSCAN components. The green points indicate core-members, yellow indicates non-core members, and red indicates outlier. The dashed lines indicate distances that are greater than threshold distance (d_max_) and the thick lines indicate distances that are with in d_max._

A second more efficient clustering approach, **D**ensity-**B**ased **S**patial **C**lustering of **A**pplications with **N**oise (DBSCAN) utilizes an additional density-based restriction on the clustering algorithm^26^. Here points are initially grouped that are closely packed together (high density areas) as clusters, with isolated points being identified as “noise”. The DBSCAN algorithm uses a *core sample* concept comprised of *core* (points close to each other by a selected distance measure) and *non-core* or *boundary/fringe* members which are close to but not a *core* (Fig. 5). The algorithm decides whether a point is a *core*, *non-core* or an *outlier* for a cluster. The maximum distance parameter (d_max_) determines the threshold distance up to which two points are considered neighbors, and the minimum sample parameter (α) determines the minimum number of data points required to define observation as core member. Based on d_max_ and α, a *core* can be defined as a point which has at least α number of points (including itself) within a distance of d_max_, while a *non-core* can be defined as a point that is not a *core* but still reachable from a *core*. A cluster needs to have least one core member and can have any number of non-core members. An outlier is a point that is not reachable from any core, even though it might be reachable from a non-core. This is the biggest difference between the two cluster types as the agglomerative cluster can grow its boundary through outlier to outlier connections.

Various components of the DBSCAN algorithm are outlined in Fig. 5. The thick black arrows indicate distances that are within the maximum distance parameter (d_max_), while the dashed arrows indicate distances that are greater than d_max_. The minimum sample parameter (α) is assigned as 5 for this case. The green colored points are core points having at least α neighbors (including itself) which are within a distance of d_max_. The yellow colored is a non-core point as it is reachable only from a single point. The red point is an outlier as it is not reachable from any core points, and only reachable from a non-core point. All the green points (core members) along with the yellow point (non-core member) form a cluster while the red point is treated as an outlier.

To run the algorithm, a random point is selected, and its neighboring points are identified using the d_max_. If the point has α number of neighbors within d_max_, then it is marked as a *core* and the cluster formation procedure is started. All the neighbors for the *core* are combined together to form a cluster and the neighbor points are further checked to determine whether they are also *cores*. If a neighbor point is also a *core* then its neighbors are also added to the current cluster increasing the total membership. After cluster formation another random unvisited point is taken and the algorithm is repeated until all points have been visited. For this and the agglomerative clustering, the date for each cluster member is recorded to allow for a simultaneously constructed longitudinal analysis of the clusters. In this way the current cluster “map” can be compared against the previous Covid situation.

## Results

GeoMEDD is the result of a research-practice partnership between university-based spatial epidemiologists and health system-based clinicians, data scientists, and operations management personnel. It has evolved into a location based early warning system for the Northeast Ohio region that is utilized daily to link early insights into early interventions. In this section, we provide example summaries of how GeoMEDD has been utilized to limit the local COVID impact. Unfortunately, specific examples (names and locations) cannot be presented due to confidentiality reasons.

### Assessing care home risk

From an early period, there was an understanding that congregate housing and especially care homes were particularly vulnerable to COVID. As a result, a list of care homes connected to the hospital system were ingested into the spatial database to became part of an automatic query whereby any new positive results in or around would be evaluated as to whether a health care “intercept” team should be mobilized. From a spatial perspective this included using a series of automatic buffers to assess positive case locations at 200, 500, 1000 and 2500 m for the prior 21 days. By reading the progression of cases across distance bands, the changing state of disease in and around the facility was checked on a daily basis. Table 1 provides an example of the 200 m buffer option which was chosen instead of a single address due to the sprawling nature, multiple name and address, and frequent geocoding variances that often accompanied each care home campus. In this example we see that Home 1 has added one additional positive test three days ago, while Home 12 registered its first positive in Time period 3. When reading this table across the full 21 days it is possible to see the emergence of localized disease curves, both at the facility level and (when considering the 2500 m banding) in the surrounding neighborhood.

**Table 1:**
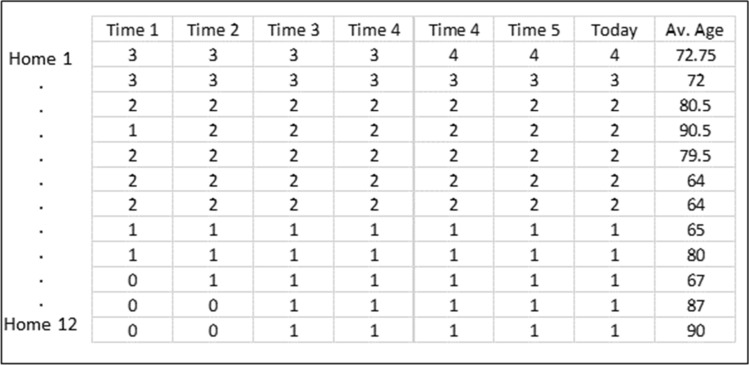
Example output table showing COVID positive test results each day within 200 m of twelve different care homes.

As the 200 m buffer is supposed to capture those living inside the facility but could still capture proximate private residences in that distance, the column of average age was used as a first filter to remove obviously spurious positive cases. The new case at Home 12, for example, is 90 years old, which suggests a care home resident.

It soon became evident that the “known” list of care homes was not comprehensive. Assisted living facilities, retirement communities, even apartment complexes marketed more to a senior living clientele were missing from the ingested list and yet also needed to be considered. The emergent *sentinel* cluster provided an invaluable means to tease out these other facilities by first considering the average age of the cluster, and then using Google Street View to literally “see” if the address was a private residence or congregate housing. Intercept teams, when appropriate, were also mobilized to these out-of-health-system buildings.

### Congregate housing and social vulnerability

**Figure 6:**
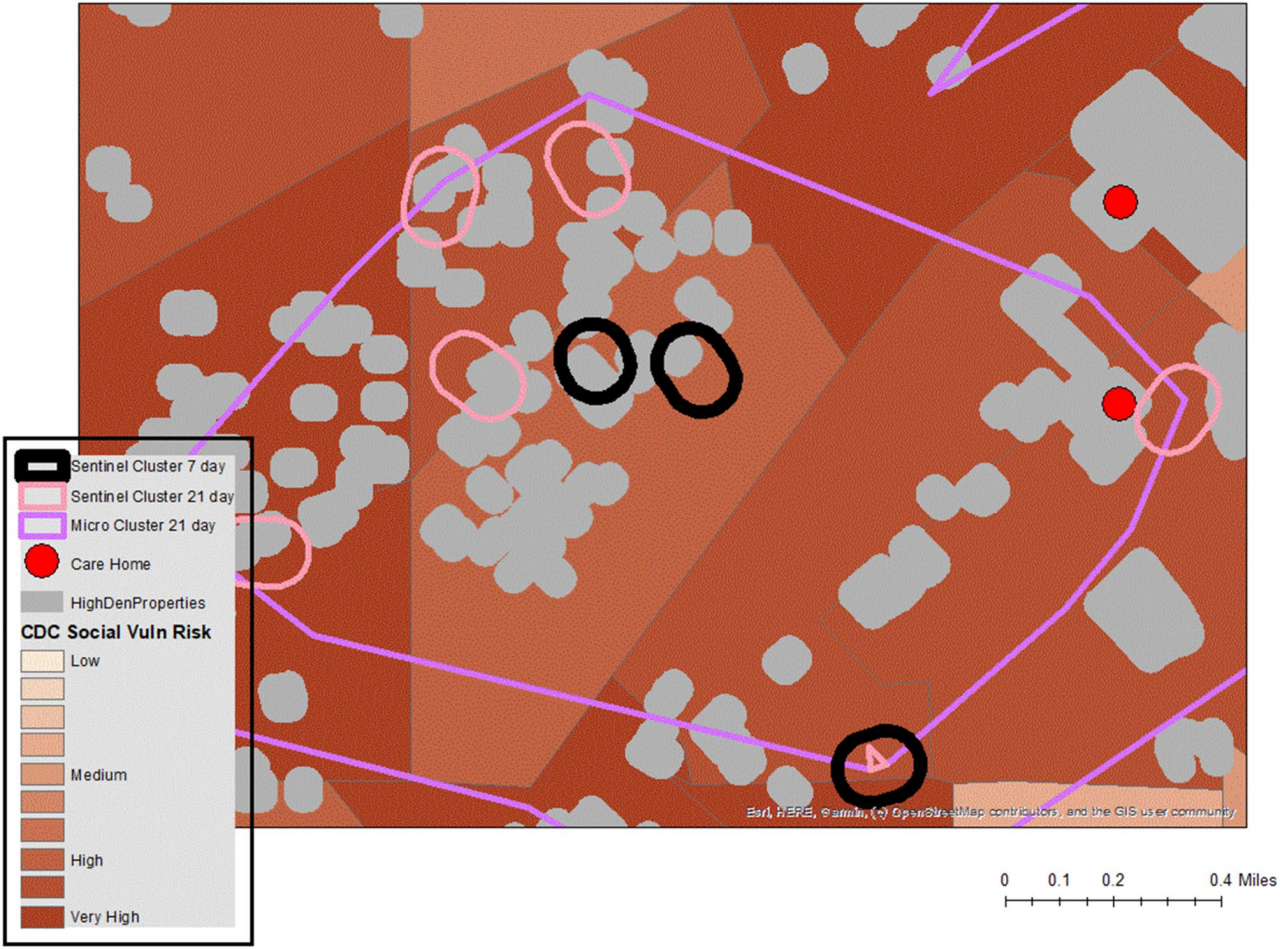
Example of micro and sentinel clusters using synthetic data.

As previously mentioned, a common finding in the United States is the disproportionate COVID toll faced by certain cohorts, especially minority populations and those living with limited financial means. Reasons include the high proportion of co-morbidities carried by this population, being in potentially high infection spaces such as essential work environments, and using public transport. There is also a higher likelihood of living in congregate housing and / or multi-generational families. Daily analysis of cluster patterns further validated these findings when clusters were mapped onto higher “socially vulnerable” areas as defined by the CDC SVI measure^23^ or by ADI^24^. Figure 6 provides an example (using synthetic data) of the type of automated cluster output that would be brought into a GIS, and then investigated for patterns. The *micro* cluster contains several 21 day *sentinel* clusters, and three 7 day emergent *sentinel* clusters. Most of these fall on congregate housing (the grey shapes), while the underlying vulnerability of the area as defined by the CDC is also displayed, with all these census tracts falling into the more vulnerable end of the spectrum. Also of note, is the proximity of one sentinel cluster close to a care home (the red dot). This would normally result in a “deeper dive” into this area looking at the nature of all positives, all pending tests, and any other related factors such as local ambulance runs.

While clustering did also occur in higher income areas, for example during the beginning phases of the pandemic when medical workers (including doctors) tested positive, clusters more consistently occurred in vulnerable neighborhoods. While there was still some temporal variation to these cluster strengths, one particular area of high vulnerability consisting of 5 connected neighborhoods produced micro clusters during every week of surveillance even as the overall disease situation fluctuated. These areas also produced a high percentage of severe cases inside clusters, and a higher percentage of cluster members carrying existing comorbidities. From an intervention perspective, when clusters emerged in congregate housing in these areas (the grey sections on the map), intercept teams were informed, local public health or city managers were notified, and in some instances mobile testing operations mobilized.

### Rural clustering

One of the biggest challenges during the early stages of the pandemic for the Northeast Ohio area was monitoring rural environments for disease intensity. Rural geographies pose multiple spatial problems, from heterogeneous population densities, to errors with geocoding addresses. Additionally, local health systems might be more resource limited than their metropolitan counterparts. From an associated data input perspective, two additional challenges were encountered, firstly traditional social vulnerability measures such as SVI tend to be less meaningful in rural environments, partly because of the large size of the census tracts encountered with dispersed population therein, and partly because the traditional input factors may not be as relevant for rural settings. Secondly, other data used to capture built environment risks, such as combining parcel types to form the “high density living” map layer seen in Fig. 6 are usually specific to local administrations, with counties or even cities being the author of these data. As a result, quality and availability is highly variable, especially in more rural counties where it is often hard to acquire such detailed building information.

During the 6 months of disease monitoring, GeoMEDD identified several rural disease clusters of which local health departments were informed or to which intercept teams were mobilized. Of particular note were local clusters centered on care homes which then spilled over into the local community. (See https://www.youtube.com/watch?v=iaJNyddZkPs&feature=youtu.be). While both *sentinel* and *micro* clusters identified outbreaks in these types of named congregate housing (care homes usually associated with a hospital through a post-acute care network), there was more of a challenge picking out other local clusters unless they occurred in the same residence. This is because “neighbors” often live more than 100 m apart on the same road. However, incorporating a spatial database means that modifying cluster dimensions is easily achievable, with one fix being a hybrid version of the *sentinel* and *micro* cluster which uses 2 members within 500 m. In this instance the agglomerative cluster is also more appropriate to add on additional cases (neighbors) rather than using local density.

### Covid risks around education

During the summer of 2020 attention expanded to whether opening educational buildings, from day cares to universities, posed a risk to society. In Ohio this decision was left to individual institutions and school districts. At the time of writing, two cluster examples in the education setting are worth noting. Firstly, a neighborhood cluster emerged that initially was believed to be fueled by a local university. Indeed, a second neighborhood cluster less than a mile away provided such an example. However, on further examination of the cluster it was largely comprised of mid-teenage males. While no contact trace was employed within the health system, it is the feeling of the analytics team that this cluster was probably due to the local high school sports team testing its athletes. Interestingly the school itself was fully remote outside of its athletic program. These results raised the possibility of community and intergenerational spread within the same home. Especially as the surrounding neighborhood was again more “vulnerable”. Currently both these questions are being explored.

The second example included a university that was not actively testing students. However, clusters (*neighborhood*, *micro* and *sentinel*) emerged in the proximate off campus areas. Further investigation found a high proportion of the positive cases were early 20 s living in either student designed congregate housing or fraternity houses. While the full picture of exposure was not available due to a lack of testing, especially given the lower numbers of severe manifestations, these clusters did provide an example of classic syndromic surveillance, where a limited dataset provides enough of a warning to mobilize greater resources, which is what then happened.

## Discussion

The continual daily surveillance of COVID has created a need to reconsider how we approach the spatially granular aspects of disease spread, in near real time. Questions arise such as is the cluster in a post-acute care home, or other congregate housing, or are there other high density living nearby? If so, is this a new cluster? Do we know about the facility or is it relatively unknown? Are there connecting paths or roads between “hot” buildings? Is there an ongoing temporal pattern to the cluster, or is there just a short burst and then stagnation? While contact tracing remains a vital part of understanding disease spread, the clusters described here reveal geographic patterns and aspects of diffusion in near real time—far in advance of other public health monitoring in the region. While more traditional methods of geographic cluster detection are still important, often their scale of operation, both temporally and spatially, is not suited for on-the-ground operations. The approach presented here has provided a solution which also lessens the burden on analytics teams who can easily become overwhelmed with the amount of inflowing data and the overall intensity of the situation.

One comment raised is that we are analyzing only one health care system. Indeed, one of the main problems working with health care data in the United States is the fragmented nature of the system. It is entirely possible that a county containing a major metropolitan center has multiple hospitals systems, as well as a city and county health department. While a complete data picture is possible at the top of the health hierarchy, for example the state health department, accessing these data, especially in a timely fashion can be challenging. However, this is a syndromic surveillance approach – it is designed to reveal patterns where there is incomplete data. In other words, the clustering may not capture the entire disease situation, it is still enough to understand the emerging nature of disease in the area. Obviously the predictive power is greatest where one health system dominates the local market, but we have found that even in more limited settings, patterns emerge that provide real time indicators of developing risk. This was no better illustrated than with the university opening example. The age structure associated with these clusters immediately revealed that the positives were all in their early 20 s. By adding in the addresses of cluster members several student congregate houses, and fraternity buildings were identified. For a university where testing was not being conducted, these clusters strongly suggest a local problem is emerging, with a possible broader community risk. Within a few days of reading this cluster landscape, the area as a whole moved into a state-designated “high risk” category based on all available test data. However, the syndromic surveillance provided by the clusters provided a few extra days of warning.

There are other limitations associated with GeoMEDD beyond incomplete data. While “trigger” visualizations are always evolving to assess when a cluster becomes deep enough (in terms of positives), big enough, or has the right context to warrant attention, there is still the need for a human interface, with the most effective being an individual with a spatial data background with experience in looking at maps and understanding their dynamism. Though GeoMEDD has reduced burden on data analysts, there is still benefit to look with an experienced eye at how spatial and temporal cluster trends change, both single clusters and more regional patterns. We would argue that over automation might even lessen the effectiveness of GeoMEDD if we lose this process of developing a “feel” for the emerging and shifting disease landscape.

Furthermore, GeoMEDD does not rely on denominator values. It is true that a basic tenant of spatial analysis is that unless patterns are normalized you could just be mapping underlying population. We emphasize that GeoMEDD does not replace traditional forms of disease cluster detection, rather it enhances investigation in near real-time and in the context of health system operations to a dynamic situation. Furthermore, this lack of requiring a reference population in GeoMEDD is not relevant here for three main reasons. Firstly, this is syndromic surveillance, so knowing where the first case(s) occur is vital to mobilize interventions – it doesn’t matter how many live in the tower apartment complex. Secondly, the importance of a denominator is more relevant for cross sectional analyses. Here clusters are being generated continuously, and assessed on a daily basis especially at the height of a disease wave. The underlying population does not change so patterns emerging on that surface have already been normalized by time. While it could be argued that a particularly dense housing area might continuously produce more clusters because of the underlying population, our experience is that cluster stability occurs because of other factors such as social vulnerability and built environment infrastructure. That is not to say that more typical spatial analysis methods are not valid, and indeed are used in tandem by our team, especially at inflection points in the pandemic as we look back and identify the patterns that only reveal themselves through longer data periods and with more time for reflection. Thirdly, what is the correct denominator? Arguably positivity rates, so therefore the amount of testing performed is more a useful measure. Unfortunately, six months into the pandemic, testing supplies are still limited, with various community testing initiatives waning accordingly. While we do incorporate positivity into each cluster to further contextualize it, we are fully aware that testing is geographically biased, and that asymptomatic cases and lower acuity cases largely go untested.

Technological limitations will also vary in terms of how data are ingested, processed and mapped using GeoMEDD. While we have championed the use of the spatial database, and this is by far the most effective means of combining and querying spatial data, allowing for the easy modification of queries (and clusters) to address new questions, and then automating outputs, not every health system has the ability to develop the code required. However, the basic concept of the clusters described here can be implemented using typical database and display architecture common place in any health system, even without a GIS. Alternatively, for other users a standard GIS can also replicate the method.

Finally, as mentioned early in this paper, while COVID provided the stimulus for these new method developments, the application extends far beyond this pandemic: from other future epidemics, to regular flu seasons, from outbreaks of overdoses to surveillance looking for bioterror or radiological symptoms. GeoMEDD should be seen as the first step in the development of a suite of clusters nuanced to varying geographies (urban versus rural, even vertical living environments), or specific topics. The spatial database and the conceptual structuring is the skeleton awaiting different bodies. And as with any method, future versions will improve upon, theoretically justify (or critique), and generally test the limits of what we have proposed here.

## Data Availability

A Scipy based working implementation for agglomerative and DBSCAN algorithm can be found in https://github.com/JayakrishnanAjayakumar/SyndromicSurveillance.
